# Reaction with Proteins of a Five-Coordinate Platinum(II) Compound

**DOI:** 10.3390/ijms20030520

**Published:** 2019-01-26

**Authors:** Giarita Ferraro, Tiziano Marzo, Maria Elena Cucciolito, Francesco Ruffo, Luigi Messori, Antonello Merlino

**Affiliations:** 1 Department of Chemical Sciences, University of Naples Federico II, 80126 Napoli, Italy; giarita.ferraro@gmail.com (G.F.); cuccioli@unina.it (M.E.C.); ruffo@unina.it (F.R.); 2 Department of Pharmacy, University of Pisa, 56126 Pisa, Italy; tiziano.marzo@unipi.it; 3 Department of Chemistry, University of Florence, 50019 Sesto Fiorentino (FI), Italy

**Keywords:** five-coordinate Pt complexes, anticancer platinum compounds, protein metal coordination

## Abstract

Stable five-coordinate Pt(II) complexes have been highlighted as a promising and original platform for the development of new cytotoxic drugs. Their interaction with proteins has been scarcely studied. Here, the reactivity of the five-coordinate Pt(II) compound [Pt(I)(Me) (dmphen)(olefin)] (Me = methyl, dmphen = 2,9-dimethyl-1,10-phenanthroline, olefin = dimethylfumarate) with the model proteins hen egg white lysozyme (HEWL) and bovine pancreatic ribonuclease (RNase A) has been investigated by X-ray crystallography and electrospray ionization mass spectrometry. The X-ray structures of the adducts of RNase A and HEWL with [Pt(I)(Me)(dmphen)(olefin)] are not of very high quality, but overall data indicate that, upon reaction with RNase A, the compound coordinates the side chain of His105 upon releasing the iodide ligand, but retains the pentacoordination. On the contrary, upon reaction with HEWL, the trigonal bi-pyramidal Pt geometry is lost, the iodide and the olefin ligands are released, and the metal center coordinates the side chain of His15 probably adopting a nearly square-planar geometry. This work underlines the importance of the combined use of crystallographic and mass spectrometry techniques to characterize, in detail, the protein–metallodrug recognition process. Our findings also suggest that five-coordinate Pt(II) complexes can act either retaining their uncommon structure or functioning as prodrugs, i.e., releasing square-planar platinum complexes as bioactive species.

## 1. Introduction

Cisplatin (*cis*-diamminedichloridoplatinum(II)) and its second-generation analogues are among the most used anticancer drugs [1,2,3]. The mechanism of action of these drugs involves a number of critical events, such as cellular uptake and transport to the nucleus, binding to nuclear DNA, and recognition by DNA-binding proteins and DNA-processing enzymes [4,5,6]. Unfortunately, like most chemotherapies, platinum antitumor drugs suffer from major drawbacks, including the development of acquired drug resistance, and nephrotoxicity, neurotoxicity, gastrotoxicity, ototoxicity, and emetogenicity [7]. Direct Pt interactions with proteins play an important role in these adverse effects [8,9,10,11,12].

To overcome these side effects, structurally and functionally unique metallodrugs have been designed [1]. These include *trans*-configured complexes [13], Pt(IV) compounds [14,15], polynuclear complexes [16], photoactivatable complexes [17], and even five-coordinate (trigonal bi-pyramidal) complexes of general formula [Pt(X)(Y)(N–N)(olefin)] (X = halide; Y = alkyl, aryl, or halide) [18,19]. Five-coordinate Pt(II) species are usually stabilized by the presence of sterically hindered nitrogen chelates, e.g., 2,9-Me_2_-1,10-phenanthroline (dmphen, Me = methyl). It has been guessed that such ligands inhibit olefin loss because the resulting square-planar product would suffer severe constraints in the coordination plane [20,21,22,23].

Five-coordinate (5C) Pt(II) complexes possess a good thermal stability, can be stored at air for a long time, and show interesting antitumor properties in vitro [18,19,23,24]. However, very little is known about the mechanism of action of these compounds, and even less is known about the reactivity with biological macromolecules, like nucleic acids and proteins. In particular, a recent paper describes the interaction of a five-coordinate platin compound with DNA [25], whereas another very recent work reports on the interaction with proteins [18]. In this latter, the X-ray structure of a five-coordinate Pt complex–protein adduct is also reported. This has been obtained by reaction of the metal compound 1Pt-Im—i.e., a five-coordinate Pt(II) compound where Pt coordination sphere comprises dmphen, an imidazole derivative containing a sugar ligand (Im), ethylene, and a methyl—with the model protein bovine pancreatic ribonuclease [18]. The X-ray structure of the adduct presents unusual features, with the Pt center retaining the trigonal bi-pyramidal (tbp) geometry and two distinct fragments that are bound to the side chain of His105 on the surface of the two molecules of RNase A present in the asymmetric unit [18]. In particular, Pt containing fragments bound to the protein include Im, dmphen, and ethylene, or dmphen, ethylene, and the methyl ligand bound to the Pt center [18].

The unusual results reported in this work prompted us to analyze, in more depth, the reaction of a five-coordinate Pt(II) compound with the model proteins hen egg white lysozyme (HEWL) and bovine pancreatic ribonuclease (RNase A) using electrospray ionization mass spectrometry (ESI MS) and X-ray crystallography.

**Figure 1:**
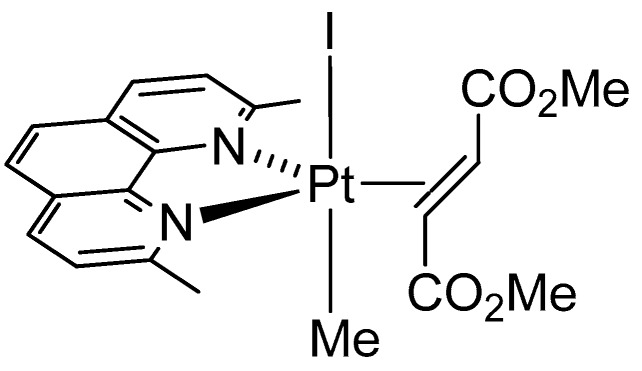
Structure of complex **I**.

Specifically, experiments are aimed at elucidating the structural bases of five-coordinate compound/protein recognition to provide possible hypotheses on the mechanism at the basis of the antitumor activity of these compounds. The five-coordinate complex [Pt(I)(Me)(dmphen)(olefin)] (Me = methyl, dmphen = 2,9-Me_2_-1,10-phenanthroline, olefin = dimethylfumarate) was synthetized and characterized by NMR, as previously reported [20]. This compound is characterized by tbp geometry of the metal center (complex **I**, Figure 1).

HEWL and RNase A have been chosen as model proteins, since they have already been used as templates to study the protein–metallodrug recognition process and, thus, unravel the molecular basis for protein–metal complexes recognition [26,27,28,29,30,31].

## 2. Results and Discussion

### 2.1. Spectroscopic Studies

**Figure 2:**
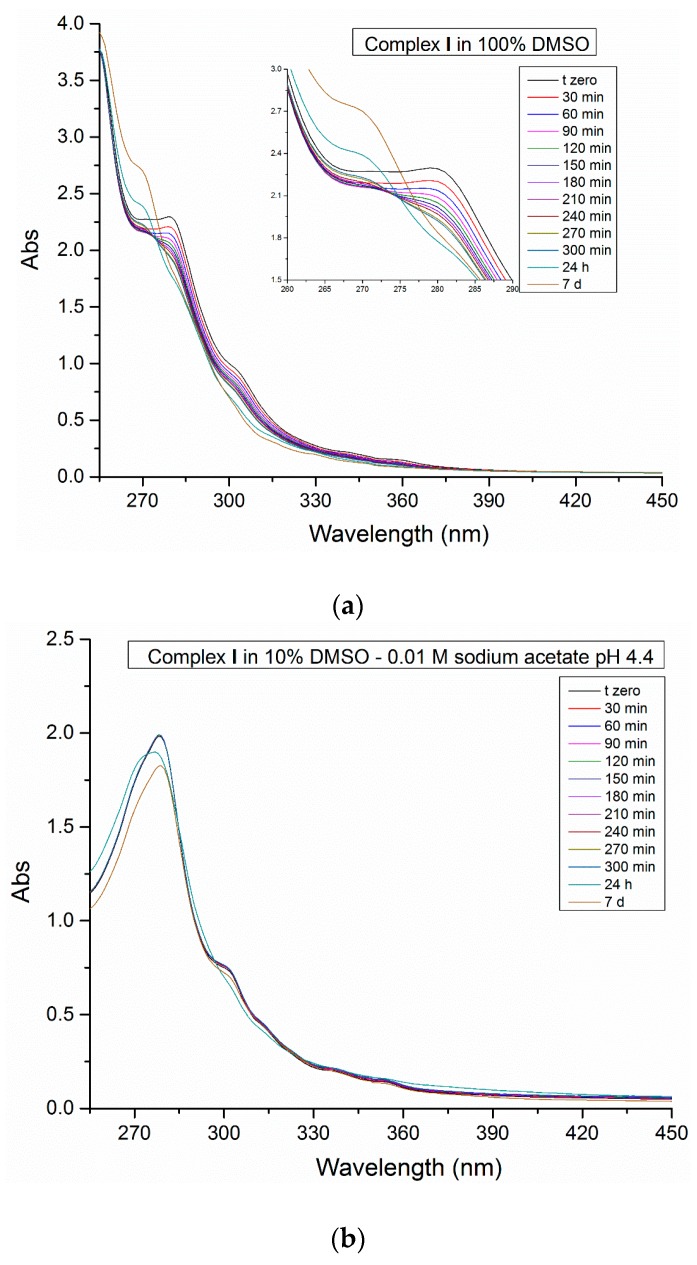

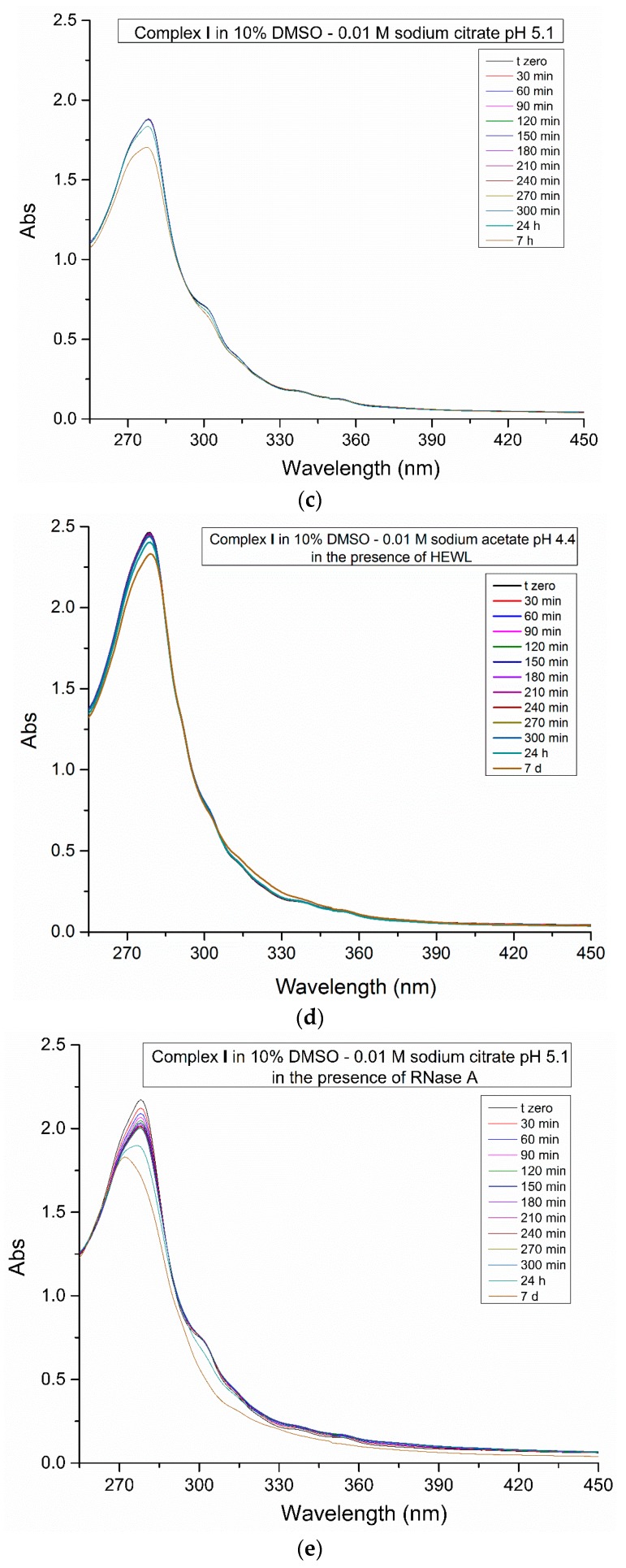
Time dependent UV–Vis absorption spectra (up to 7 days) of 5 × 10^−3^ M complex **I** in 100% DMSO (**a**), 90% DMSO–0.01 M sodium acetate buffer pH 4.4 (**b**), 90% DMSO–0.01 M sodium citrate buffer pH 5.1 (**c**). For the last two conditions, time-dependent UV–Vis absorption spectra have been registered also in the presence of hen egg white lysozyme (HEWL) (**d**) and bovine pancreatic ribonuclease (RNase A) (**e**), respectively.

To investigate the stability of complex **I** in DMSO and in reference buffers at different pH values, a spectrophotometric study was carried out. The initial spectrum of complex **I** is characterized by a strong absorption at about 279 nm and a shoulder at 304 nm. The π→π* bands at 304 nm could be attributed to metal-ligand charge transfer (MLCT) transitions, in agreement with previous literature data [32,33]. The monitoring of the electronic spectra over 7 days shows significant changes in the absorption profile when the complex **I** is dissolved in 100% DMSO (Figure 2A). There is a progressive decrease of the Pt compound absorbance and a band blue shift from 279 to 269 nm, which are probably related to a ligand exchange process that occurs after several hours of incubation. The ligand exchange reaction is further confirmed by the superimposition of the spectra, which reveals the appearance of an isosbestic point. This behavior has been verified also by using NMR proton spectroscopy. Slow substitution of the nitrogen bidentate ligand by DMSO and simultaneous release of the alkene was observed, with formation of a square-planar Pt(II) species with Me still bound to the Pt center (Figure S1). Contrarily, complex **I** appears stable in mixed DMSO/aqueous solutions, and retains its tbp geometry under different environmental conditions, including that used to soak the compound in HEWL and RNase A crystals (Figure 2b,c).

Later on, the same process was monitored spectrophotometrically in the presence of the proteins (Figure 2d,e); in these cases, the spectra of the adduct formed in the reaction between the proteins and complex **I** show a slight decrease of intensity of the shoulder at 304 nm, although it should be noted that in these experiments the main absorption peak of complex **I** is overlaid to that of the protein, making it difficult to detect any change in the electronic structure of the metal complex by UV–Vis absorption spectroscopy.

### 2.2. Crystallographic Studies

**Figure 3:**
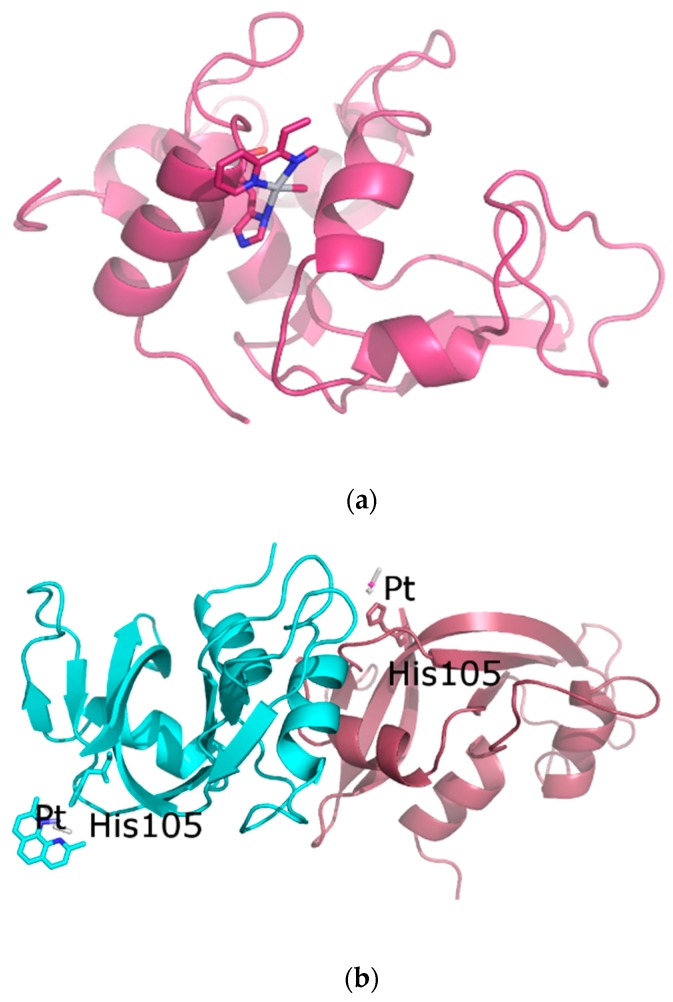
Overall structures of the HEWL (**a**) and the RNase A adducts (**b**). The Pt center is bound to the side chain of His15 in the HEWL adduct, and to the side chains of His105 of both RNase A molecules in the asymmetric unit of the crystal of the RNase A adduct.

Crystallographic data were then collected to characterize the adducts formed upon reaction of complex **I** with the two model proteins HEWL and RNase A (Figure 3a,b).

#### 2.2.1. Structure of the HEWL Adduct

Crystals of HEWL suitable for X-ray diffraction studies were grown in 20% ethylene glycol, 0.6 M NaNO_3_, and 0.01 M sodium acetate buffer at pH 4.4, and soaked in a solution prepared by adding complex **I**, freshly dissolved in DMSO, to the same reservoir (final metal complex concentration about 5 mM). The final protein-metallodrug ratio is estimated to be about 1:5. X-ray diffraction data have been collected at 100 K without cryoprotectant, following the procedure often used to collect data on protein-metallodrug adduct crystals [34,35,36]. The structure was solved at 1.96 Å resolution and refined up to R-factor of 16.8 (R-free 20.4) (see Table S1 for details).

The molecular structure of the adduct formed in the reaction between HEWL and complex **I** (HEWL adduct) is shown in Figure 3a. The overall HEWL conformation is not significantly affected by the reaction with complex **I**: the root mean square deviation (rmsd) of the positions of the carbon alpha (CA) atoms in the adduct from the native coordinates (PDB code 193L) is as low as 0.23 Å.

**Figure 4:**
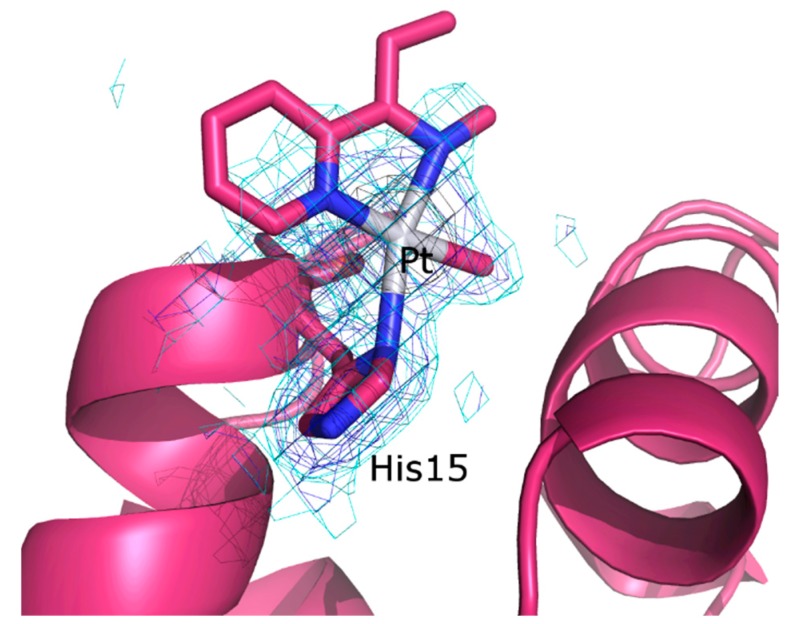
Details of the binding site of complex **I** fragment in the HEWL adduct. According to our interpretation of the electron density (e.d.) maps, binding of complex **I** fragment to HEWL is associated with the loss of two Pt ligands, i.e., the iodide, which is probably replaced by the imidazole of the His15 and the olefin. 2Fo–Fc electron density maps are contoured at 3σ (black), 1 σ (grey), and 0.5 σ (cyan) level. The electron density map associated to dmphen is poorly defined; thus, only a part of this ligand has been included in the final model. The 2Fo–Fc map contoured at 0.3σ level is reported in Figure S2.

Pt is bound to the ND1 atom of His15, to a monoatomic ligand, interpreted as the C of the Me, and to two other atoms from different molecules or from a bidentate ligand. We have interpreted the electron density map, adding part of dmphen to the model. The Pt center in the metal-containing fragment adopts a distorted, square-planar geometry (Figure 4). The olefin and the iodide ligand are not present in the final structure. In the binding site, the metal center and its ligands have an occupancy factor equal to 0.50 (B-factor for Pt atom = 49.9 Å^2^). The electron density map of the dmphen ligand is not well defined, although it clearly indicates that, in this region, a bulky Pt ligand, like dmphen, is bound to the metal center. In fact, the refinement of a model which included two monoatomic ligands according to the square planar geometry does not improve R and Rfree when compared to our interpretation, and leaves significant peaks of electron density in the Fo–Fc map. Refinement of the whole dmphen ligand with occupancy factor of 0.50 leads to atomic coordinates with B-factors between 41.9 Å^2^ and 50.4 Å^2^. The analysis of residual peaks in the Fo–Fc electron density map and the refinement of the B-factor corresponding to C atom of the Me group suggest that this ligand is, at least in part, replaced by a heavier atom. These results could indicate that in the crystal state, alternative modes of binding for complex **I** fragment(s) are possible within the His15 site.

His15 has been already found to be the Pt-binding site in many structures of adducts formed in the reaction of HEWL with Pt complexes [37,38,39,40,41]. Notably, in the present case, only the ND1 atom of His15 is involved in the recognition of the metal, contrary to what found in the case of cisplatin, which can bind both ND1 and NE2 atoms of His15, and even a nitrogen atom of the side chain of Arg14 [39,40]. This finding suggests that a Pt-containing fragment bulkier than cisplatin is bound to the side chain of His15, indirectly supporting our interpretation of the map.

#### 2.2.2. Structure of the RNase A Adduct

**Figure 5:**
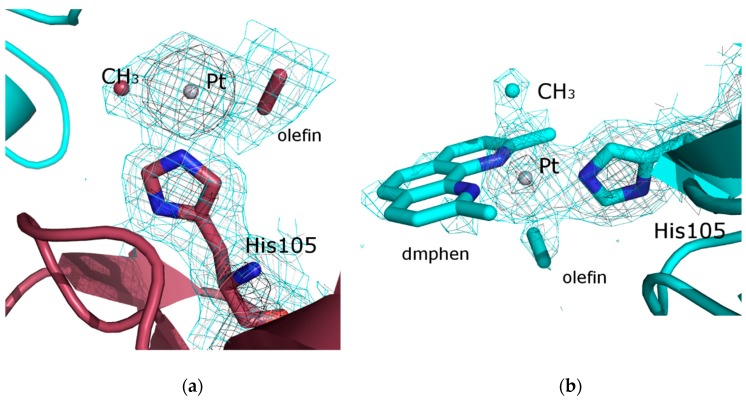
Details of the binding sites of complex **I** fragments in molecules A (**a**) and B (**b**) of the RNase A adduct. Binding of complex **I** fragments to RNase A is associated with the loss of Pt ligands, but the scarce definition of the electron density maps does not allow for an undoubtful assignment to be made. 2Fo–Fc electron density maps are contoured at 3σ (black), 1 σ (grey), and 0.5 σ (cyan) level. 2Fo–Fc electron density map of the binding site of molecule B is contoured at 0.3σ level in Figure S3.

The adduct formed upon reaction of RNase A with complex **I** was obtained by the soaking procedure on crystals of wild-type protein that were been grown in 22% PEG4000 and 0.01 M sodium citrate buffer at pH 5.1. In particular, RNase A crystals were soaked in a solution prepared by adding complex **I**, freshly dissolved in DMSO, to the same reservoir (final metal complex concentration about 5 mM). The final protein–metallodrug ratio was estimated to be about 1:5. X-ray diffraction data have been collected at 100 K without cryoprotectant. The structure was solved at 2.03 Å resolution and refined up to an R-factor of 17.6 (R-free 25.4) (see Table S1 for details). Secondary and tertiary structures of the two molecules of RNase A (A and B, hereafter) present in the asymmetric unit (a.u.) are not affected by the Pt compound binding (Figure 3b): CA rmsd with respect to the two molecules in the asymmetric unit of ligand free protein (PDB code 1JVT) are within the range 0.37–1.36 Å. The inspection of the electron density maps reveals the presence of Pt complex fragments close to His105 in both molecules of the a. u. (Figure 5), as observed in the structure of RNase A in complex with 1Pt-Im [18] and in the case of other RNase A–Pt-based drug adducts [42,43]. The interpretation of the electron density map at the two different Pt-binding sites is rather difficult. In molecule A (Figure 5a), we prefer not to assign all ligands around Pt, although residual electron density is observed in the Fo–Fc e.d. map close to the metal center. Our model contains only a Pt center bound to Me and a part of the olefin molecule (Figure 5a). Attempts to fit the residual map with the dmphen ligand led to superimpositions with atoms of residue 22 of a symmetry-related molecule. Attempts to obtain a clearer e.d. map using additional X-ray diffraction datasets failed: data have been collected on the same protein adduct from different crystals, but the e.d. maps still remain unclear.

In molecule B, it is possible that Pt retains its tbp geometry, with the His105 imidazole bound to a [Pt(Me)(dmphen)(olefin)]^+^ fragment (Figure 5b). This is in agreement with the result obtained when the structure of the adduct of RNase A with 1Pt-Im has been solved [18]. The different behavior of molecules A and B in the a.u. of the RNase A adduct is not surprising, since it is in line with previous studies [44,45], and with the finding that the soaking procedure [36] allows the protein chains in the crystal to react with complex **I** at different times.

### 2.3. Mass Spectrometry Studies

**Figure 6:**
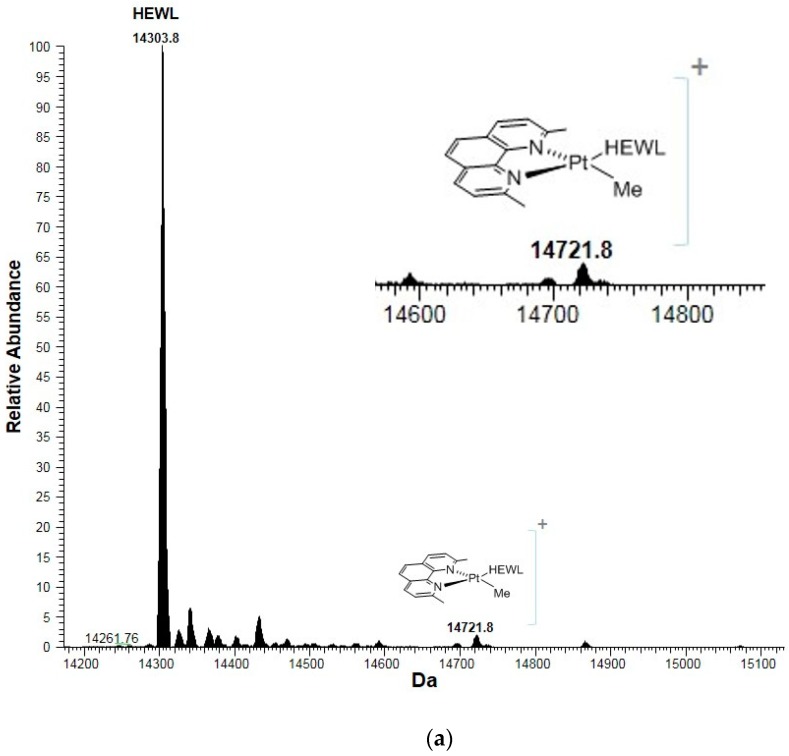

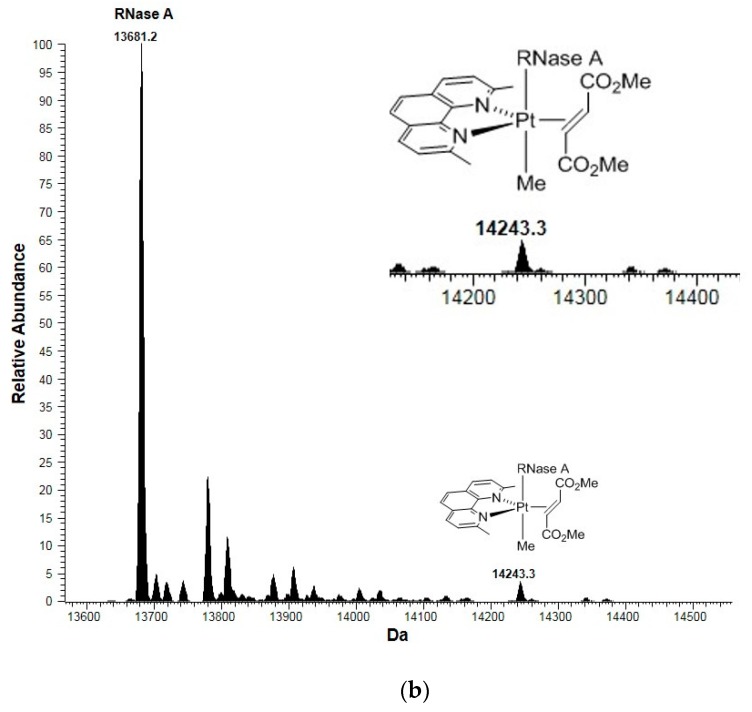
Deconvoluted ESI MS of HEWL (**a**) and RNase A (**b**) treated with 10^−4^ mol L^−1^ complex **I** (metal–protein ratio = 3:1) in 20 mmol L^−1^ ammonium acetate buffer, pH 7.0) recorded after 72 h of incubation at room temperature.

To clarify the doubts on the ligand assignment coming from the X-ray structures of the RNase A adduct and of the HEWL adduct, high resolution electrospray ionization mass spectra (ESI MS) were collected (Figure 6a,b). The analysis of the ESI MS spectra indicate that complex **I** poorly reacts with both proteins.

Interestingly, ESI MS data also reveal that when the five-coordinate compound is incubated with the two model proteins, different results are obtained. In particular, upon 72 h of complex **I** incubation with RNase A, a small new peak is detected at 14,243.3 Da, which corresponds to an adduct formed by the bovine protein with the fragment [Pt(Me)(dmphen)(olefin)]^+^. On the contrary, when complex **I** reacts with HEWL under the same experimental conditions, a small new peak is detected at 14,721.8 Da, which corresponds to an adduct formed by HEWL with a fragment of the type [Pt(Me)(dmphen)]^+^. These findings suggest that the Pt compound can bind to proteins retaining the five-coordinate Pt center or after extensive degradation concomitant to the release of the I^−^ and olefin ligands. These findings have been further validated by theoretical simulations of MS peaks reported in Figures S4 and S5.

Overall, these data, combined with the crystallographic results, could have interesting implications for the comprehension of the behavior of bioactive five-coordinate Pt(II) compounds. These molecules could interact with proteins (and maybe also with nucleic acids), retaining their tbp geometry. However, they could also change metal coordination geometry upon reaction with proteins, releasing the ligand(s) responsible for the stability of the 5C; the resulting square-planar Pt compounds can then act like cisplatin and cause tumor cell death.

## 3. Materials and Methods

### 3.1. Materials

Complex **I** was synthetized as previously described [20] and characterized by ^1^H NMR. Hen egg white lysozyme (HEWL), bovine pancreatic ribonuclease (RNase A), and all other chemicals were purchased from Sigma-Chemical Co and used without further purifications.

### 3.2. Spectrophotometric Data

The in-solution stability of complex **I** was tested by UV–Vis absorption spectroscopy under the following experimental conditions: 100% DMSO, 90% DMSO–0.01 M sodium acetate buffer pH 4.4, 90% DMSO–0.01 M sodium citrate buffer pH 5.1, in the absence and in the presence of HEWL and RNase A using a Pt compound concentration of 5 × 10^−3^ M and a protein to metal molar ratio of 1:5. The UV–Vis spectra have been collected o<n a Varian Cary 5000 UV–Vis–NIR spectrophotometer at room temperature using a quartz cuvette with 1 cm path length and the following parameters: 250–450 nm wavelength range, 200 nm/min scanning speed, band width 1 nm, data pitch 1 nm. The UV–Vis spectra were continuously registered every 30 min for 5 h and after 24 h and 7 days of incubation.

### 3.3. Crystallization, X-ray Diffraction Data Collection

HEWL and RNase A crystals were prepared by the hanging drop vapor diffusion method at 298 K in a 24 well Linbro plate. HEWL crystals were obtained using 0.5 μL HEWL solution (15 mg mL^−1^) mixed with 0.5 μL reservoir and equilibrated against 500 μL reservoir solution containing 20% ethylene glycol, 0.6 M NaNO_3_, and 0.01 M sodium acetate pH 4.4, whereas RNase A crystals were obtained by mixing the protein at 20 mg mL^−1^ with an equal volume of reservoir containing 20% PEG 4000 and 0.01 M sodium citrate at pH 5.1.

Crystals of the adducts have been obtained by soaking native protein crystals in a solution prepared by adding complex **I**, freshly dissolved in DMSO, to the same reservoir (final metal complex concentration about 5 mM). The final protein-metallodrug ratio is estimated to be about 1:5, whereas the amount of DMSO in the final solution is 25%.

X-ray diffraction data have been collected at the CNR Institute of Biostructures and Bioimages, Naples, Italy at 100 K using a CCD Saturn 944 detector and a Cu rotating anode. X-ray diffraction data of the HEWL adduct and of the RNase A adduct have been collected at 1.96 Å and 2.03 Å resolution, respectively, using an oscillation range of 1 deg, exposure time of 5 s, and a crystal to detector distance of 45 mm. The crystals have been maintained at 100 K by the Oxford Cryostream system without cryoprotectant, following the procedure already used to collect diffraction data on crystals of other protein–metallodrug adducts [36]. The obtained data have been processed using the HKL2000 package [46]. Data collection statistics are reported in Table S1.

### 3.4. Structure Solution and Refinement

The structures of the HEWL adduct and of the RNase A adduct have been solved with program Phaser [47] and refined with Refmac5 [48]. Coordinates of proteins used as starting models are taken from PDB codes 193L [49] and 1JVT [44]. Inspection of the initial electron density maps clearly reveals the presence of a Pt atom bound to His15 in the case of HEWL, and close to His105 in the case of RNase A. The occupancy of the Pt atoms has been evaluated, applying many refinement steps for different fixed values of the occupancy, until the lower residual positive and negative peaks of electron density map were observed at the Pt-binding sites. In the case of HEWL, after refinements including the Pt atom, large positive peaks of electron density appeared close to the metal center. These peaks have been attributed to the methyl group and to some atoms of the dmphen ligand. Since the electron density map of the dmphen ligand is not so well defined, we have tried to verify this assignment by analyzing the residual Fo–Fc e.d. map corresponding to the coordination sphere of the Pt center obtained after the inclusion of just three atoms (C of the methyl and two oxygens) in the classical square planar geometry. However, after refinement of the model including just three atoms in the planar square geometry close to Pt, positive peaks of electron density appear. This suggests the presence of a bulkier ligand, like dmphen, close to the metal center. The assignment also well agrees with the results of ESI MS. In the case of RNase A, we have preferred to not assign the Pt ligands in molecule A, whereas a [Pt(Me)(dmphen)(olefin)]^+^ fragment was modeled in molecule B. This is in agreement with ESI MS spectra.

The final structures of the HEWL and RNase A adducts have been refined up to R-factor and Rfree values of 16.8%/17.6% and 20.4%/25.4%, respectively. The coordinates and the structure factors have been deposited into the PDB with the codes 6QEA and 6QE9.

### 3.5. Electrospray Ionization Mass Spectrometry

RNase A and HEWL (10^−4^ M) were incubated at room temperature (25 ± 1 °C) for 72 h with complex **I** (3:1 metal–protein ratio) in 0.020 M ammonium acetate pH 7.0. After a 20-fold dilution with water, ESI MS spectra have been recorded by direct introduction at 5 μL min^−1^ flow rate in an Orbitrap high-resolution mass spectrometer (Thermo, San Jose, CA, USA), equipped with a conventional ESI source. The working conditions were the following: spray voltage 3.1 kV, capillary voltage 45 V, capillary temperature 220 °C, tube lens voltage 230 V. The sheath and the auxiliary gases were set, respectively, at 17 (arbitrary units) and 1 (arbitrary units). For acquisition, Xcalibur 2.0. software (Thermo) was used and monoisotopic and average deconvoluted masses were obtained by using the integrated-Xtract tool. For spectrum acquisition, a nominal resolution (at *m/z* 400) of 100,000 was used.

## 4. Conclusions

In conclusion, we have studied the reaction of a five-coordinate Pt(II) compound with two model proteins through a combined ESI MS/X-ray crystallography approach [50,51].

Our experiments show that complex **I** can react with proteins, leading to different products. Although the quality of the X-ray structures of the HEWL and RNase A adducts is not very high, the combination of the crystallographic results and mass spectrometry data allow drawing the following picture: when complex **I** reacts with HEWL, a Pt-containing fragment binds at His15 side chain. Upon binding, complex **I** extensively degrades, losing the iodide and olefin ligands from the metal center, and probably changing its coordination geometry from tbp to square-planar. On the other hand, when the Pt compound reacts with RNase A, the Pt-containing fragment formed upon releasing the iodide ligand binds close to the side chain of His105.

Based on these results, it is possible to speculate that pentacoordinate Pt(II) compounds with tbp geometry can react with proteins either retaining their unusual structure or releasing square-planar Pt compounds.

## Supplementary Materials

Supplementary Materials can be found at http://www.mdpi.com/1422-0067/20/3/520/s1.

## Abbreviations

5C	pentacoordinate
a.u.	asymmetric unit
CA	carbon alpha
cisplatin	*cis*-Pt(NH_3_)_2_Cl_2_
dmphen	2,9-dimethyl-1,10-phenanthroline
DMSO	dimethylsulfoxide
DNA	deoxyribonucleic acid
e.d.	electron density
ESI MS	electrospray ionization mass spectrometry
HEWL	hen egg white lysozyme
Im	imidazole
Me	methyl
MLCT	metal–ligand charge transfer
NMR	nuclear magnetic resonance
olefin	dimethylfumarate
PDB	Protein Data Bank
PEG	polyethylene glycol
tbp geometry	trigonal bi-pyramidal geometry
rmsd	root mean square deviation
RNase A	bovine pancreatic ribonuclease
UV–Vis	ultraviolet–visible
