# Supplementary material for: Reaction with Proteins of a Five-Coordinate Platinum(II) Compound
Source: Int J Mol Sci. 2019 Jan 26;20(3):520. doi: 10.3390/ijms20030520 (PMC6387405; doi:10.3390/ijms20030520)
Supplement: Supplementary file 1 [file ijms-20-00520-s001.pdf]

International Journal of
Molecular Sciences

MDPI

Supplementary Information

Reaction with Proteins of a Five-Coordinate Platinum(II) Compound

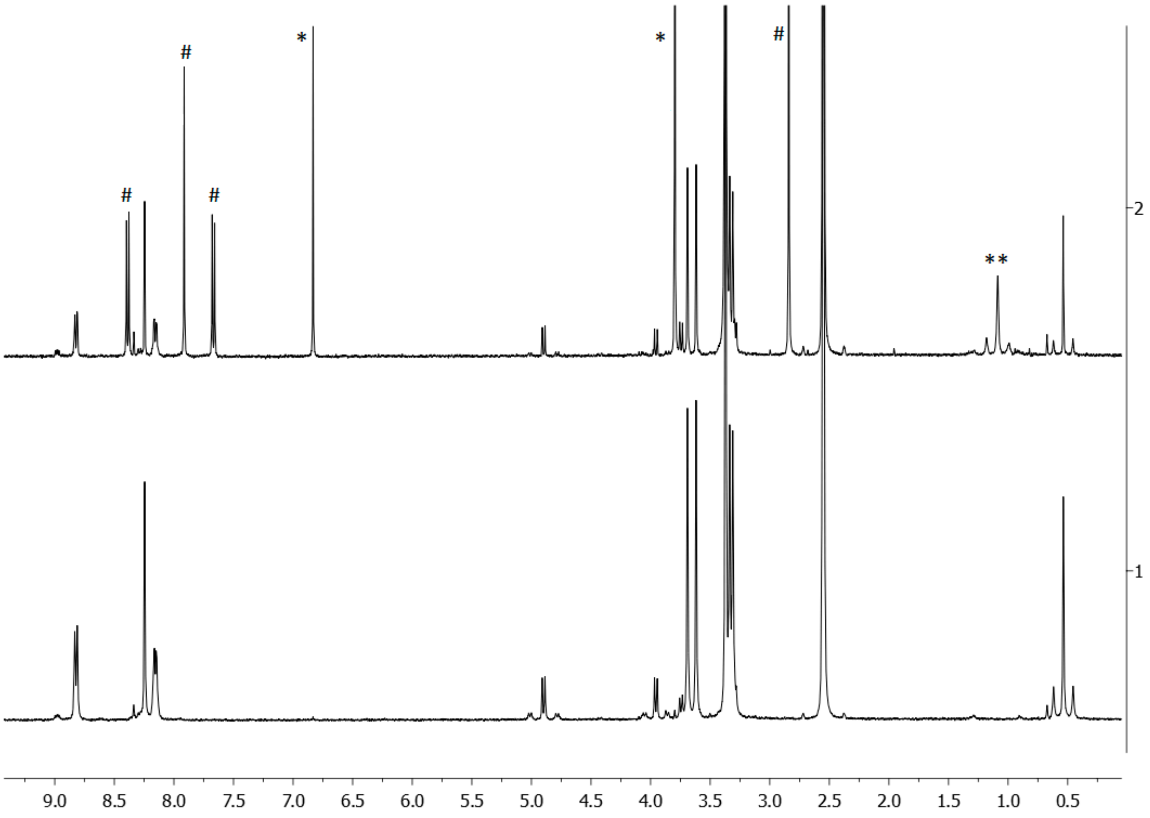

Figure S1. ^1H NMR spectra at 400 MHz in DMSO-d_6 of [Pt(I)(Me)(dmphen)(dimethylfumarate)] after 5 minutes (trace 1) and after 18 hours (trace 2). Legend: *: free dimethylfumarate; #: free dmphen; **: Pt-Me in the square-planar product.

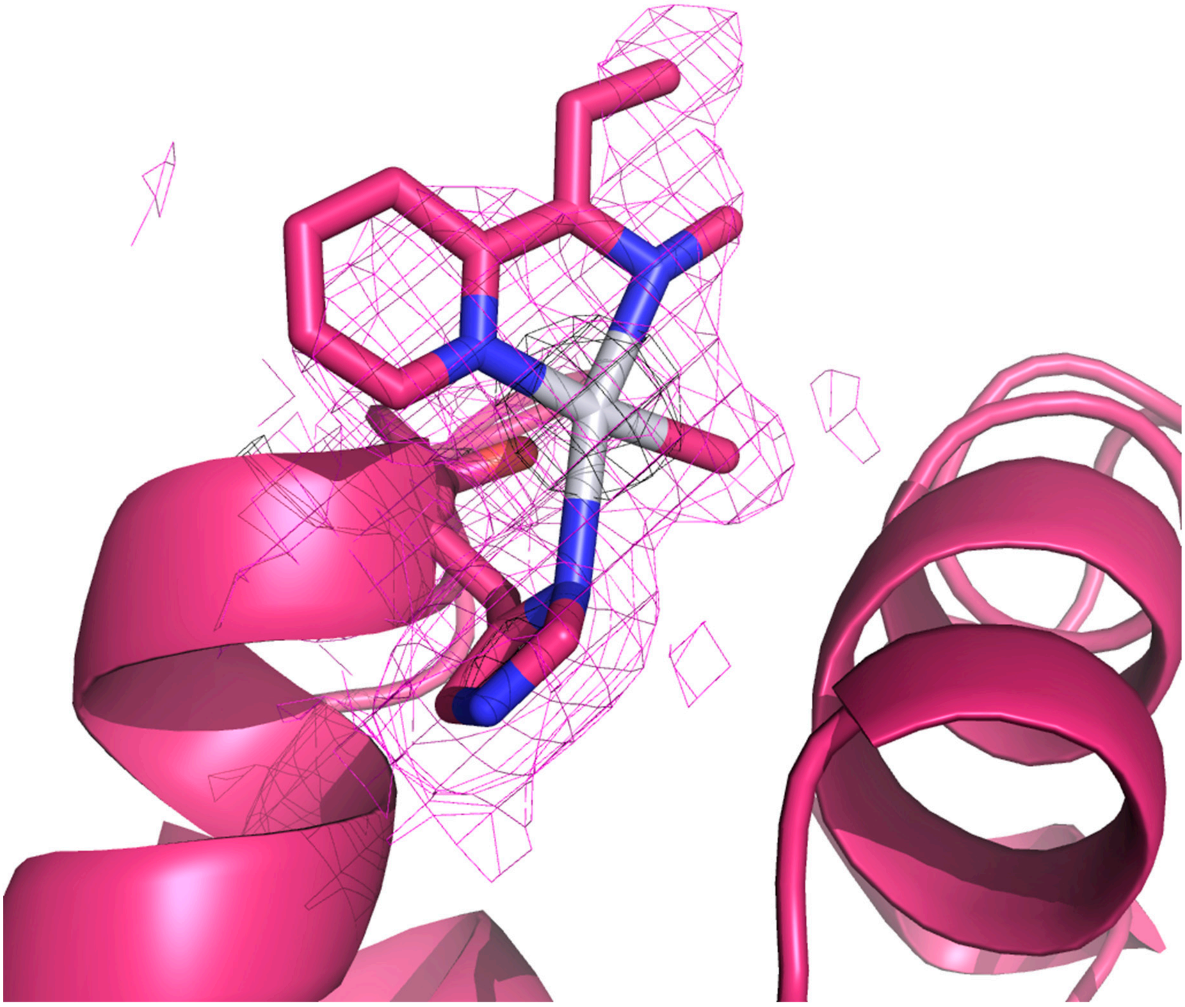

Figure S2. Details of the binding site of compound **I** fragment to HEWL. 2Fo-Fc electron density map is contoured at 3σ (dark grey), 0.3 σ (purple) level. Pt is in grey.

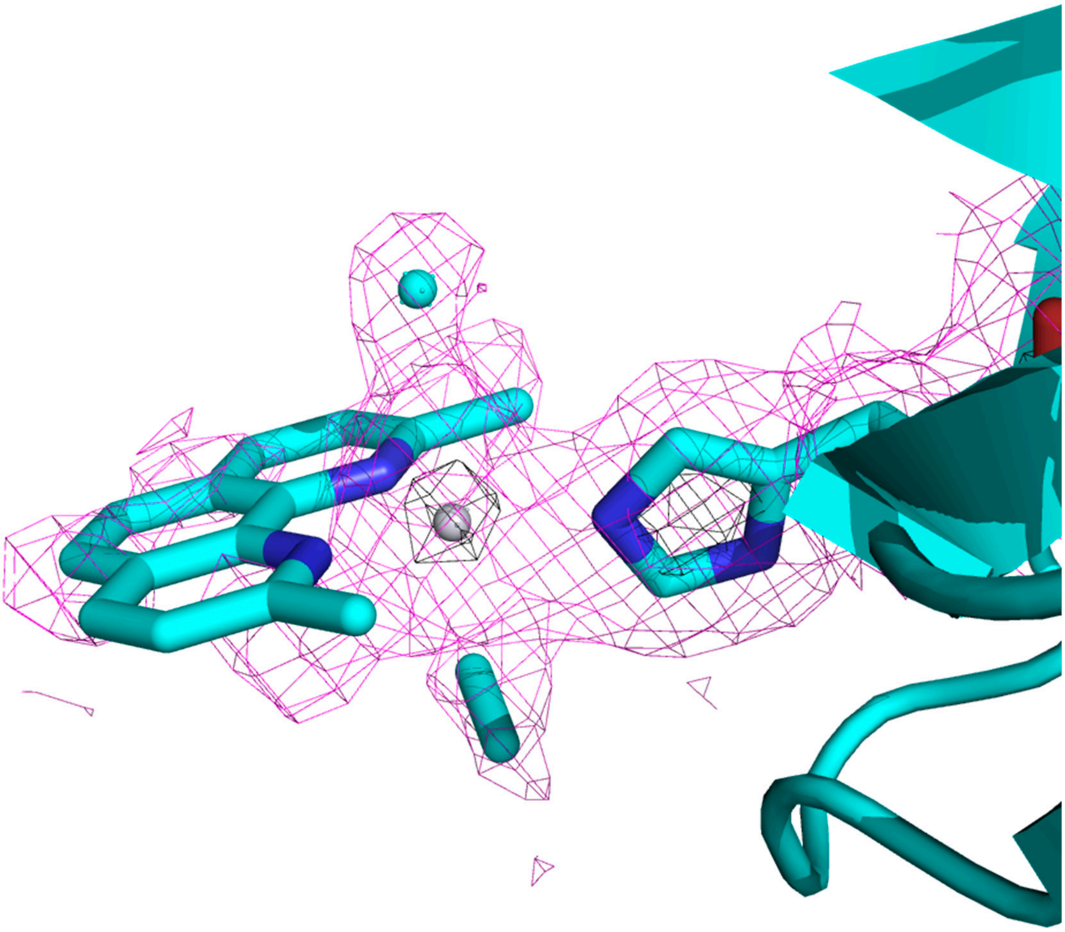

Figure S3. Details of the binding site of compound **I** fragment to molecule B of RNase A-compound **I** adduct. 2Fo-Fc electron density maps are contoured at 3σ (dark grey) and 0.3σ (purple) level. Pt is in grey.

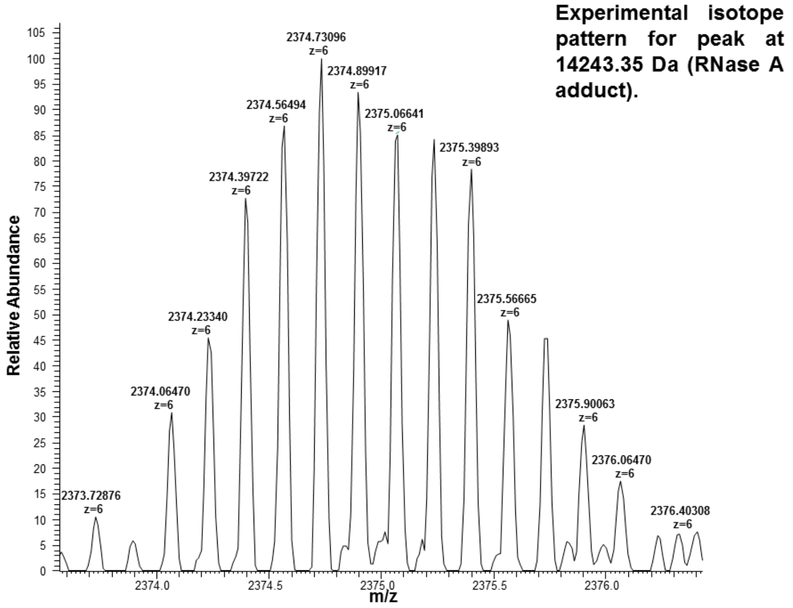

(a)

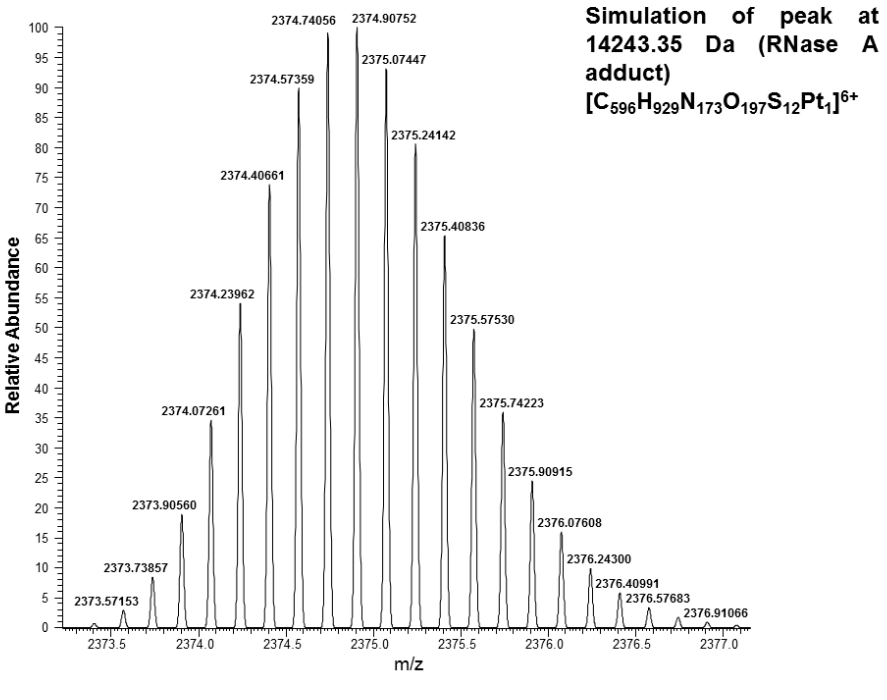

(b)

Figure S4. Experimental isotope (a) and theoretical simulation (b) pattern for the peak at 14243.35 Da obtained when RNase A was incubated with complex **I** (RNase A adduct)

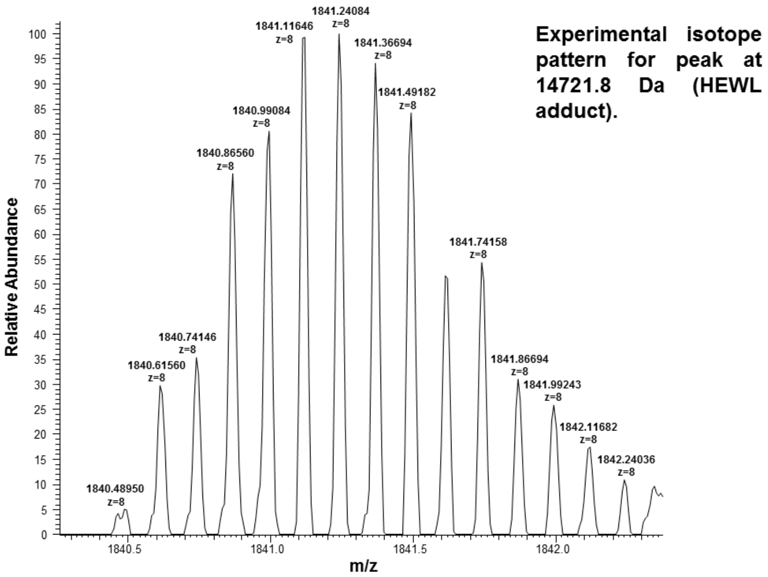

(a)

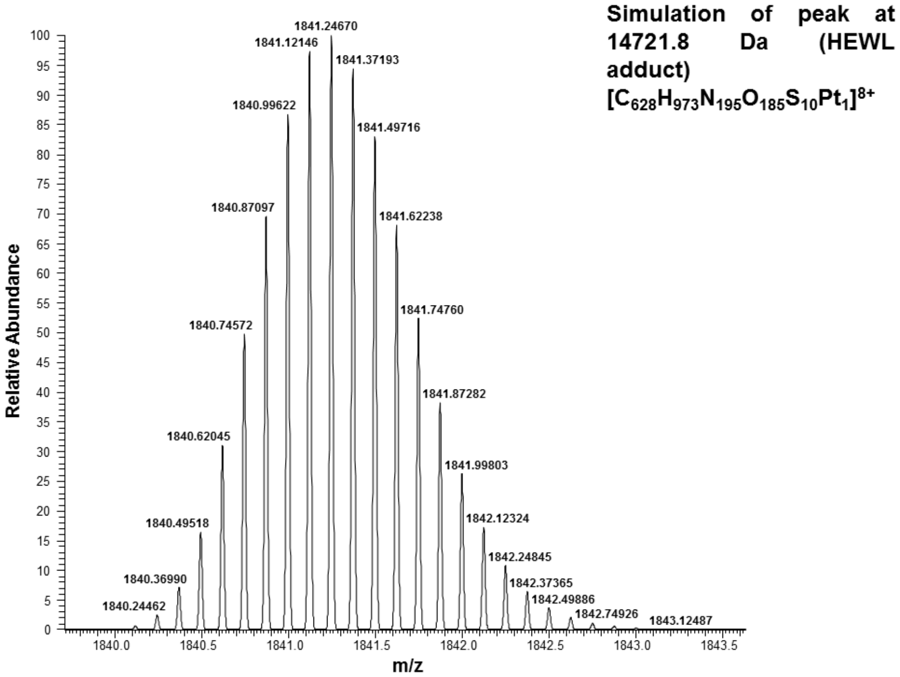

(b)

Figure S5. Experimental isotope (a) and theoretical simulation (b) pattern for the peak at 14721.8 Da obtained when HEWL was incubated with complex **I** ((HEWL adduct)

Table S1. Data collection and refinement statistics

	HEWL-Compound **I**	RNase A-Compound **I**
PDB code	6QEA	6QE9
Data-collection		
Space group	$P4_32_12$	C2
Unit cell parameters		
a,b,c (Å); β	78.36, 78.36, 37.35; 90.0	99.00, 30.66, 68.68; 93.0
Molecules per asymmetric unit	1	2
Observed reflections	58658	36001
Unique reflections	8815	12949
Resolution (Å)	55.41-1.96 (1.99-1.96)	50.00-2.03 (2.07-2.03)
Rmerge†	0.099 (0.597)	0.085 (0.528)
Completeness (%)	99.9 (100.0)	94.8 (92.3)
I/σ(I)	13.3 (2.9)	8.9 (2.3)
Multiplicity	6.7 (5.0)	2.8 (2.8)
Refinement		
Resolution (Å)	55.41-1.96	22.89-2.03
number of reflections in working set	8367	12313
number of reflections in test set	417	636
R factor/Rfree (%)	0.168 (0.204)	0.176 (0.254)
Number of non-hydrogen atoms	1178	2088
Occupancy of Pt ion	0.50	0.60, 0.40
B-factor of Pt ion (Å^2)	49.9	40.0, 74.7
Overall B-factor (Å^2)	23.0	30.8
Ramachandran values (%)		
Most favoured/ Additional allowed	96.5/ 3.5	94.9/3.0
Generously allowed/ Disallowed	0.0	2.1*
R.m.s.d. bonds (Å)	0.009	0.008
R.m.s.d. angles (Å)	1.62	1.52

*Outliers are Gln60B, residues of the molecule A hinge peptide (residues 18, 19 and 23), which is disordered in many structures of RNase A including RNase A-compound I, and Ser90B.
